# Improving influenza vaccination rates during COVID-19 pandemic – the need of the hour

**DOI:** 10.7189/jogh.11.03042

**Published:** 2021-04-17

**Authors:** Harshil Bhatt

**Affiliations:** 1 Goshen Hospital, Goshen, Indiana, USA; 2 Indiana University School of Medicine, South Bend, Indiana, USA

Coronavirus disease-2019 (COVID-19), caused by the severe acute respiratory distress syndrome coronavirus-2 (SARS-CoV-2), started as an outbreak of pneumonia in Wuhan, China, in December 2019 and on March 11, 2020, the world health organization (WHO) declared it as a pandemic. Since then, COVID-19 has affected millions of people all around the world and has caused significant disruption of our physical, mental, and social health. COVID-19 vaccine is still a work in progress, and currently, social distancing and facemasks are the only few measures being used to prevent the spread of the virus.

While the northern hemisphere is on the verge of embracing the fall season, it means the next flu season seems not too far as well. According to a study by The Center for Disease Control and Prevention (CDC) and global health partners in 2017, up to 646 000 people die worldwide from influenza-related illnesses every year, and up to 72 000 of these deaths occur in Europe. The estimates of mortality from influenza have significantly gone up in the last few years [1]. Unfortunately, the flu season is going to be much more different and challenging this time due to the presence of COVID-19. It could lead to a substantial increase in morbidity and mortality.

The major difference, however, between influenza and COVID-19 is that we already have a vaccine available for the former. Influenza vaccines can prevent influenza infections and can also decrease the rate of hospitalizations and deaths among the recipients, especially young children, older adults (>65 years), and pregnant women. As per the CDC estimates, more than 35.5 million cases, nearly 490 600 hospitalizations, and 34 200 deaths were due to influenza during the 2018-2019 flu season in the United States [2]. Also, during the same 2018-2019 season, the influenza vaccination rate was approximately 49%, which, according to the CDC, prevented 4.4 million influenza illnesses, 58 000 hospitalizations, and around 3500 deaths [3]. It is quite clear that during the time of COVID-19, low influenza vaccination rates during the 2020-21 flu season could make these numbers significantly worse. This could seriously damage the efforts that are under way to control the COVID-19 pandemic by increasing health care demand and exhausting the available resources. This could further jeopardize and weaken adequate health care delivery.

But it has been a struggle to improve the influenza vaccination rate. Questions have always been raised about the effectiveness of the flu vaccine. It is estimated that the effectiveness of a flu vaccine is generally around 40%-60% when the circulating viruses are well-matched [4]. Moreover, certain myths prevail and adversely affect vaccination rates. “One can catch flu from a flu vaccine,” “healthy persons do not need flu shots,” or even, “flu is not a serious disease” are just some of the examples of such false beliefs.

## CREATING AWARENESS ABOUT INFLUENZA VACCINATION

One question that could come to the mind of an epidemiologist, a health policymaker, or a health professional is if COVID-19 has created enough awareness that it could help improve the seasonal influenza vaccination rates.

A survey of 10 957 adults in the United States between April 29 to May 5, 2020, showed that at least 28% of the adults knew someone who was diagnosed with COVID-19 and around 20% of them knew at least one person who had been hospitalized or even died due to COVID-19 [5]. Every day, there is plenty of new information that comes out regarding COVID-19, and it is not wrong to believe that the COVID-19 pandemic might have created awareness among the people, and that can prove to be useful when it comes to getting the flu vaccination. It is not hard to imagine that people are likely to be more receptive during this time of the pandemic, regarding influenza, its risks including hospitalization and pneumonia, and benefits of vaccination.

Is it safe, though, to assume that the psychological effects of the COVID-19 pandemic could help the way of clearing doubts among people who were reluctant to get a flu vaccine during the previous flu seasons?

## STRATEGIES TO IMPROVE INFLUENZA VACCINATION RATES

Healthcare policymakers and health care professionals could come together and strategize a systemic approach to create awareness about the importance of flu vaccine. Social media and digital media platforms could prove useful for running campaigns regarding the benefits of getting vaccinated during the time of the COVID-19 crisis. The general population should be explained about the myths surrounding the vaccines. A particular focus should be on the vulnerable population as well, such as children, older adults, and pregnant women.

**Figure:**
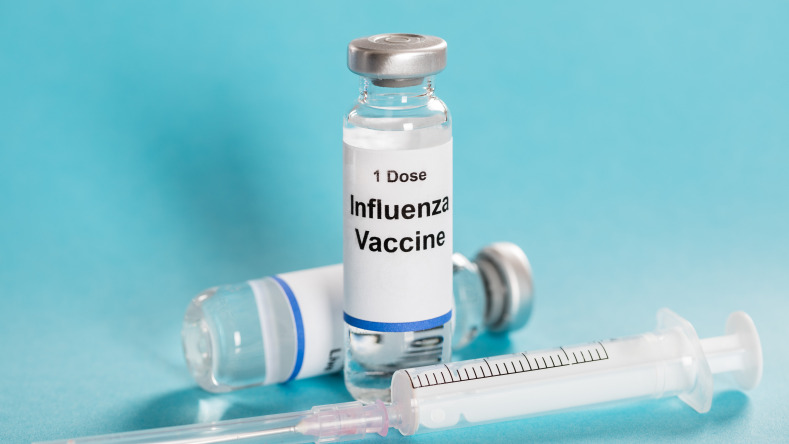
Photo: By Andrey Popov for Depositphotos (used with permission).

Directing efforts towards improving health care access for safe vaccine administration should also be a priority. Since during the time of COVID-19 pandemic, health care providers are more likely to conduct virtual visits via telehealth service, especially for regular physicals or annual visits, that platform could be utilized to remind the patients about flu vaccination. However, limiting clinic visits could decrease flu immunization rates as well. It must be ensured that safe access is created for patients to get flu shots. It is not difficult to organize a “drive-through” for patients to receive the flu vaccine, the similar way by which many clinics have executed plans for COVID-19 testing. “Flu vaccination drive” or such events could be arranged with proper safety precautions (masks, temperature checks, sanitizers) at certain businesses, workplaces, nursing homes, hospitals, schools, colleges, or places of worship if they remain open during this time. Help from local outpatient pharmacies could be undertaken to accomplish such vaccine drives. Healthcare policymakers should also look into creating mandates for requiring flu vaccinations at a broader level or even at smaller scale targeting health care facilities, nursing homes, schools, or essential businesses. It should be ascertained that the vaccine availability and production are at par to meet the increase in demands. Vaccine costs should not become an obstacle for those individuals who are in economic hardship due to the COVID-19 situation.

## CAN WE SUCCEED IN PREVENTING ANOTHER EPIDEMIC?

We are eagerly waiting for the COVID-19 vaccine because, in addition to the social distancing and use of masks to prevent the spread of the virus, a vaccine is something that can restore normalcy across the world. A survey of over 10 000 adults by Pew Research Center showed that around 72% of adults said that they would definitely or probably get COVID-19 vaccine when it comes out [6], Based on the same rationalization, it should not be difficult to convince people to get the flu vaccine. On a positive side, the efforts that we put for improving flu vaccination at this time will surely be equally helpful when the COVID-19 vaccine rolls out in the future.

In the era of the socio-digital world, it is not hard to believe that a process such as creating awareness about flu vaccination cannot gain momentum. Generous participation of people, the dedication of health care professionals, and the political will of policymakers are all it may take to prevent another epidemic during this time! Improving influenza vaccination rates will not only improve morbidity and mortality related to influenza but will also spare the already overburdened health system in the time COVID-19 and maintain its proper functioning without exhausting the resources. While our eyes are on the researchers to come out with a COVID-19 vaccine rather quickly, it should not look too difficult for all of us, who have experienced a lot in the last few months, to try and prevent another epidemic with a simple and safe vaccine. This is not the time to ask, “If or when can we act?” but rather “How and now!”

## References

[1] Seasonal flu death estimate increases worldwide. Center for Disease Control and Prevention. December 13, 2017. Available: https://www.cdc.gov/media/releases/2017/p1213-flu-death-estimate.html. Accessed: 14 August 2020.

[2] Estimated influenza illnesses, medical visits, hospitalizations, and deaths in the United States — 2018–2019 influenza season. Center for Disease Control and Prevention. January 8, 2020. Available: https://www.cdc.gov/flu/about/burden/2018-2019.html. Accessed: 15 August 2020.

[3] 2018–19 influenza Illnesses, medical visits, hospitalizations, and deaths averted by vaccination. Center for Disease Control and Prevention. January 16, 2020. https://www.cdc.gov/flu/about/burden-averted/2018-2019.htm. Accessed: 15 August 2020.

[4] Vaccine effectiveness: how well do the flu vaccines work? Center for Disease Control and Prevention. January 3, 2020. Available: https://www.cdc.gov/flu/vaccines-work/vaccineeffect.htm. Accessed: 15 August 2020.

[5] Johnson C, Ferno J, Keeter S. Few U.S. adults say they’ve been diagnosed with coronavirus, but more than a quarter know someone who has. Pew Research Center. May 26, 2020. Available: https://www.pewresearch.org/fact-tank/2020/05/26/few-u-s-adults-say-theyve-been-diagnosed-with-coronavirus-but-more-than-a-quarter-know-someone-who-has/. Accessed: 15 August 2020.

[6] Thigpen CL, Funk C. Most Americans expect a COVID-19 vaccine within a year; 72% say they would get vaccinated. Pew Research Center. May 26, 2020. Available: https://www.pewresearch.org/fact-tank/2020/05/21/most-americans-expect-a-covid-19-vaccine-within-a-year-72-say-they-would-get-vaccinated/. Accessed: 15 August 2020.

